# The I-CAM-FR: A French Translation and Cross-Cultural Adaptation of the I-CAM-Q

**DOI:** 10.3390/medicines5030072

**Published:** 2018-07-06

**Authors:** Leo Druart, Nicolas Pinsault

**Affiliations:** 1 School of Physiotherapy, Grenoble-Alpes University Hospital, 38000 Grenoble, France; leo.druart@hotmail.com; 2 ThEMAS Team, TIMC-IMAG Laboratory, UMR CNRS-UGA, 5525 Grenoble, France; 3 Critical Thinking Research Federation, University of Grenoble-Alpes, FED, 4270 Grenoble, France

**Keywords:** CAM, I-CAM-Q, I-CAM-FR, translation, cross-cultural adaptation

## Abstract

**Background:** The use of complementary and alternative medicine (CAM) is growing every year. The extent of its use is still not clear, and it is difficult to undertake comparative studies due to the variety of data collection tools used. Therefore, a standardized International Complementary and Alternative Medicine Questionnaire (I-CAM-Q) has been recommended to determine data about its usage. The purpose of the present study is to present a controlled translation of the questionnaire into French which is also properly adapted to a French audience. **Methods:** The English-written questionnaire, the I-CAM-Q, was submitted to three independent translators. Each translator produced a separate French translation (FT.1.1, FT.1.2, FT.1.3) that was then synthesized into a unique new version (FT.2.0). Version FT.2.0 was then submitted to three new translators, who translated them back to three distinct English versions (BT.1.1, BT.1.2, BT.1.3). These versions were once again synthesized in a unique questionnaire (BT.2.0). The version BT.2.0 was then submitted to an expert committee that compared it to the original I-CAM-Q in order to review the process and adapt FT.2.0 according to differences between the I-CAM-Q and BT.2.0. This led to a revised French version, PT.0.0. Version PT.0.0 was then tested with the use of cognitive interviews. These interviews allowed a final adjustment of the translations to produce a definitive version in French: the I-CAM-FR. **Results:** Four French translations and four versions in English of the I-CAM-Q questionnaire were produced. This allowed us to present a consolidated French translation to an expert committee. Their adjustments were taken into account before testing the final French-translated questionnaire on a group of people (*n* = 10) representing a diverse sample of the French population. The expert committee then suggested changes according to the errors due to the translation process sought out by the pre-tests, and recommendations based on the errors that were not due to the translation process. **Conclusions:** Through a rigorous methodology, we produced a French translation and a cross-cultural adaptation of the I-CAM-Q questionnaire. This work has led to the creation of an equivalent questionnaire available for use in France, the I-CAM-FR.

## 1. Introduction

Complementary and alternative medicine (CAM) is increasingly used worldwide [1,2,3]. The data available are not yet reliable due to their high variability [4,5,6,7] depending on the study and methodology used. Furthermore, in France, data about CAM usage exists mainly for specific groups of the population such as patients with chronic diseases [8]. However, in other countries, it has been demonstrated that these groups of the population tend to have a higher rate of CAM usage than the general population. France not being an exception, it seems reasonable to assert that we cannot extrapolate the results of this specific class of the population to the general population.

In 2012, the CAMbrella initiative published a literature review about CAM usage in Europe [3]. This review was unable to include France due to lack of data. In addition, they also studied the feasibility of an International Complementary and Alternative Medicine Questionnaire (I-CAM-Q) to evaluate CAM usage and to be able to compare results between countries [9,10]. The outcome of that study was that the I-CAM-Q [11] was the recommended tool and as such should be translated into other languages as soon as possible [12]. Since then, a few translations have already been performed, including German [13], and Argentinean versions [14], as well as Italian, Spanish, Dutch, and Romanian translations [9].

This paper aims at adding a French translation to the already existing versions of the I-CAM-Q.

## 2. Materials and Methods

The I-CAM-Q is an auto-administered questionnaire that aims to collect data on CAM usage. It is divided into four different sections that each evaluate a different modality of CAM use. The first section of the questionnaire focuses on the different providers consulted by participants. The second section then looks at CAM treatments prescribed or administered by the subject’s physician. The third section focuses on phytotherapy and food supplements used by the subject. Finally, the fourth section deals with self-help practices. To achieve the goal of creating a consistently translated and cross-culturally adapted French version of the I-CAM-Q, we used the following methodology. It was performed according to published guidelines [15,16,17,18,19,20,21,22]. Its production consisted of four steps.

### 2.1. Inclusion Criteria for Translators

Six different translators were selected and recruited according to the following criteria:

- They had to speak fluently in the language from which they translated.
- They had to have lived at least one full year in the country of the source language of the translation (to assure bilingualism [22,23]).
- Their mother tongue had to be the language towards which they translated.
- They had to have at least the country’s equivalent of an A level in the United Kingdom or the baccalaureate in France.

These criteria allowed us to select only capable translators that were knowledgeable in translation and cross-cultural adaptation, thanks not only to their bilingualism but also to their understanding of the cultural background of the source language.

### 2.2. Forward Translation

This step aimed at producing a first French version of the questionnaire. To achieve this, three different translators separately translated the document. Once this was done, the three versions (FT.1.1, FT.1.2, and FT.1.3) were compared. Discussions took place amongst the translators and the coordinator of the study about the differences in each translation. These discussions led to the creation of a French version of the I-CAM-Q called the FT.2.0.

### 2.3. Back Translation

The aim of this step was to translate the French version created previously back to English. With the same methodology as in the forward translation, three new translators with no previous knowledge of the I-CAM-Q independently worked on the FT.2.0. Discussions then took place between them to reach a consensus on the final English version (BT.2.0) produced during this stage.

### 2.4. Expert Committee

During this phase, the goal was to adjust the French version (FT.2.0) according to the differences reported between the I-CAM-Q and the BT.2.0. This allowed the unveiling of inexact translations.

The expert committee was comprised of one of the translators of the forward translation, one of the back translation, the study’s coordinator, a healthcare expert in the field of CAM, and an expert in research methodology. The two latter experts had also been consulted while elaborating the methodology presently used.

This committee had access to every translation (FT.1.1, FT.1.2, FT.1.3, FT.2.0, BT.1.1, BT.1.2, BT.1.3, and BT.2.0) as well as the discussions that led to the versions FT.2.0 and BT.2.0. They also helped write the introduction of the questionnaire as had been suggested and done during the elaboration of the German translation of the I-CAM-Q [13]. Given the feedback from the German team, an adapted landscape layout was used, similar to the one suggested by the German team.

Once the FT.2.0 was adjusted according to the different suggestions of the expert committee the amended resulting version (PT.0.0) was used in a pre-testing phase.

### 2.5. Pre-Testing

The pre-test’s aim was to shed light on the cognitive process that develops while people fill out the questionnaire. This allows us to test the comprehension of the translated questions and verify equivalence with the original intent of the questionnaire. To do this, cognitive interviews [24] were used. This process has been proved useful in improving the quality of questionnaires [25] and more particularly in health-related studies [26], as well as in CAM-related surveys [27].

We interviewed 10 people, creating a sample of individuals with a broad use of CAM spread out over the age spectrum. The interviewees were people who volunteered after social media demand for volunteers. Some were familiar with CAM, some were heavy users of CAM, and others had never used them. This ensured the testing of the translation covered as many cases as the pre-test allowed.

To encourage people to speak their mind, the interviews were preceded by the phrase “I did not elaborate this questionnaire so do not hold back when speaking your mind. It will not upset me, quite the contrary since the better the feedback the better the quality of the translation”.

Interviews were recorded after collecting signed consents. The questionnaires were then handed out and the interviewees were asked to read out loud the questions and explain what they understood of the question and then say and write down their answer while giving an oral justification. The interviewer then wrote down, on a separate questionnaire, the expected answer, given the oral justification. Comparisons between the questionnaire filled by the interviewer and the interviewee were then carried out.

The differences noted were then categorized according to the reason behind it: Was the error due to a misunderstanding generated by the translation? In contrast, was the misunderstanding due to the original question? According to the errors listed, the expert committee suggested changes for the errors due to translation, and listed recommendations for the errors due to the original questionnaire.

This methodology resulted in the final French translation of the I-CAM-Q: the International Complementary and Alternative Medicine questionnaire in French (I-CAM-FR).

## 3. Results

Our pre-test was conducted on a sample of 10 people. The youngest participant was aged 15 and the oldest was aged 78. The mean and median are similar, indicating the extreme values did not have too much of a one-sided influence on the sample. Four out of 10 participants were male, and all the interviewees had their main residence in an urban environment.

Changes made to the introduction of the questionnaire mainly concerned grammar.

Some errors were recurrent and present on multiple pages of the questionnaire. These errors were often related with the time frame of a question that was not taken into account (the time frame “in the last 3 months” used in certain questions was not seen by most of the interviewees for example). For these errors, we suggested modifying the layout so as to highlight better the elements in the questions that the surveyed people had overlooked.

The first page of the questionnaire showed very few errors and misunderstandings due to the translation. The second page had the largest number of errors both due to translation and due to the questionnaire in itself. The bulk of errors was explained by a misunderstanding of what this part of the questionnaire probed. Only CAM delivered by health care professionals was to be considered; however, many interviewees answered regardless. The layout of the page was reviewed, and it was decided that the introduction would insist more heavily on this nuance in the questionnaire. Other issues raised in the second page of the questionnaire included not knowing what was delivered by the therapist and not knowing exactly what a “manipulation” was. Only errors due to the original questionnaire were found on the third page, and modifications could not be made. Finally, the last part showed errors due to personal interpretation of some questions. For example, subjects did not consider their practice as meditation or prayer, while others did, even though they were sometimes identical. Also, sports and reading were often included in this part of the questionnaire.

## 4. Discussion

The aim of this study was to produce a definitive French translation and cross-cultural adaptation intended for use in France of the I-CAM-Q.

The translation of the questionnaire has nonetheless raised a few questions related to its validity. These issues are not new: several of them have already been discussed in other published studies. In 2011, Eardley et al. [10] evaluated the face validity, acceptability, and participant’s comprehension of the I-CAM-Q. They concluded that the I-CAM-Q “has low face validity and low acceptability and is likely to produce biased estimates of CAM use if applied in England, Romania, Italy, The Netherlands or Spain”. Other research articles found good face validity for the I-CAM-Q, for example that written by Quandt et al. [27].

A hypothesis that could explain these validity problems is that the questionnaires were designed to collect four types of data: data about the CAM used, data about frequency of use, data about reason of use, and data about perceived usefulness of CAM. This makes studying the validity of the questionnaire and improving it more difficult.

Other authors have also discussed the utility of epidemiological data collection relative to general CAM usage [28]. Indeed, there is a plethora of therapies, products, and therapists that fall within the scope of CAM and this category is very heterogeneous. This is illustrated in our pre-test when subjects chose to report reading and sports activities in a CAM questionnaire. Therefore, more precision is needed before use of the questionnaire relative to what data we choose to investigate and for what reasons, in order to be precise and effective. Defining more accurately the wide spectrum of CAM evaluated when using this questionnaire will also allow better sorting of the variety of answers that will result.

After having gathered data about CAM usage and before being able to compare the data from different countries it is important to consider the differences regarding CAM in these countries. As stated in the report from CAMbrella [29,30,31], the legal status of CAM varies greatly between different countries even within the European Union, where there is a common basis for some laws and regulations. This is also true for their place in the different societies and cultures. For example, the prevalence of use of acupuncture in a country where acupuncture-only therapists exist and the prevalence of use of acupuncture in a country such as France where acupuncture can only legally be practiced by medical doctors, midwives, or dentists will probably vary. Therefore, the way of interpreting the situations that the data is supposed to represent will need to take this into account if data comparison is used to answer a specific question. In the case of a consultation with an acupuncture-only therapist, the patient knows he will receive acupuncture treatment. In France, in the case of a consultation with a healthcare professional, acupuncture is but an option amongst others. This implies that the patients do not necessarily know in advance that acupuncture will be a suggested treatment option. Therefore, interpreting the difference in prevalence of use in acupuncture between these two cases may be the result of different sociological phenomena.

Therefore, after translating the I-CAM-Q, it seems its correct use will allow the gathering of data about CAM usage, and as with any data, the analysis of said data will need to take into account a plethora of factors influencing this prevalence.

## 5. Conclusions

A French translation of the I-CAM-Q now exists. It is a faithful translation of the original questionnaire. However, the inherent problems of the I-CAM-Q make its use difficult. Its merit lies in the variety of translations already achieved, allowing us to get closer to a standardized tool for the collection of data relative to CAM usage. 

In order to pursue the objective of collecting epidemiological data on products and therapies that are classified as “complementary and alternative”, we also recommend, as already voiced by other authors, focusing research on specific products and uses of CAM where we have indirect proof of spread: for example, increase of interest in the media, other studies, professional organizations such as unions, and quantity of scholarships, etc.

## References

[1] Barnes P.M., Powell-Griner E., McFann K., Nahin R.L. (2004). Complementary and alternative medicine use among adults: United States, 2002. Adv. Data.

[2] Barnes P.M., Bloom B., Nahin R.L. (2008). Complementary and alternative medicine use among adults and children: United States, 2007. Natl. Health Stat. Rep..

[3] Lewith G., Eardley S., Bishop F.L., Prescott P., Brinkhaus B., Santos-Rey K., Vas J., von Ammon K., Hegyi G., Dragan S. (2012). CAM Use in Europe—The Patients’ Perspective. Part I: A Systematic Literature Review of CAM Prevalence in the EU.

[4] Astin J. (1998). Why patients use alternative medicine? Results of a national study. JAMA.

[5] Astin J.A., Marie A., Pelletier K.R., Hansen E., Haskell W.L. (1998). A review of the incorporation of complementary and alternative medicine by mainstream physicians. Arch. Intern. Med..

[6] Gray C.M., Tan A.W.H., Pronk N.P., O’Connor P.J. (2002). Complementary and Alternative Medecine Use among Health Plan Members: A Cross-Sectional Survey. Eff. Clin. Pract..

[7] Thomas K., Nicholl J., Coleman P. (2001). Use and expenditure on complementary medicine in England: A population-based survey. Complement. Ther. Med..

[8] Morandini C. (2010). La Place des Médecines Complémentaires chez les Patients sous Chimiothérapie: Etude Prospective Multicentrique Réalisée Auprès des Patients et des Professionnels de santé de Cancérologie dans 4 Hôpitaux de la Région Rhône-Alpes. Master’s Thesis.

[9] Lewith G., Eardley S., Bishop F.L., Cardini F., Santos-Rey K., Jong M.C., Ursoniu S., Dragan S., Hegyi G., Uehleke B. (2012). CAM Use in Europe—The Patients’ Perspective. Part II: A Pilot Feasibility Study of a Questionnaire to Determine EU Wide CAM Use.

[10] Eardley S., Bishop F.L., Cardini F., Santos-Rey K., Jong M.C., Ursoniu S., Dragan S., Hegyi G., Uehleke B., Vas J. (2012). A pilot feasibility study of a questionnaire to determine european union-wide CAM use. Res. Complement. Med..

[11] Quandt S.A., Verhoef M.J., Arcury T.A., Lewith G., Steinsbekk A., Kristoffersen A.E., Wahner-Roedler D.L., Fonnebo V. (2009). Development of an International Questionnaire to Measure Use of Complementary and Alternative Medicine (I-CAM-Q). J. Altern. Complement. Med..

[12] Fischer F., Lewith G., Witt C.M., Linde K., Ammon K., Cardini F., Falkenberg T., Fønnebø V., Johannessen H., Reiter B. (2014). A research roadmap for complementary and alternative medicine-what we need to know by 2020. Res. Complement. Med..

[13] Lo Re M., Schmidt S., Güthlin C. (2012). Translation and adaptation of an international questionnaire to measure usage of complementary and alternative medicine (I-CAM-G). BMC Complement. Altern. Med..

[14] Esteban S., Vàzquez Peña F., Terrasa S. (2016). Translation and cross-cultural adaptation of a standardized international questionnaire on use of alternative and complementary medicine (I-CAM-Q) for Argentina. BMC Complement. Altern. Med..

[15] Guillemin F., Bombardier C., Beaton D. (1993). Cross-cultural adaptation of health-related quality of life measures: Literature review and proposed guidelines. J. Clin. Epidemiol..

[16] Beaton D.E., Bombardier C., Guillemin F., Ferraz M.B. (2000). Guidelines for the process of cross-cultural adaptation of self-report measures. Spine.

[17] Brislin R.W. (1970). Back-translation for cross-cultural research. J. Cross-Cult. Psychol..

[18] Brislin R.W., Lonner W.J., Thorndike R.M. (1973). Cross-Cultural: Research Methods.

[19] Brislin R.W. (1986). Research instruments. Field Methods in Cross-Cultural Research.

[20] Jones P.S., Lee J.W., Phillips L.R., Zhang X.E., Jaceldo K.B. (2001). An adaptation of Brislin’s translation model for cross-cultural research. Nurs. Res..

[21] Bouletreau A., Chouanïère D., Fontana J., Wild P. (1999). Concevoir, Traduire et Valider un Questionnaire. A Propos d’un Exemple, EUROQUEST. http://lara.inist.fr/handle/2332/1730.

[22] Sousa V.D., Rojjanasrirat W. (2011). Translation, adaptation and validation of instruments or scales for use in cross-cultural health care research: A clear and user-friendly guideline. J. Eval. Clin. Pract..

[23] Deyo R.A. (1984). Pitfalls in measuring the health status of Mexican Americans: Comparative validity of the English and Spanish Sickness Impact Profile. Am. J. Public Health.

[24] Shafer K., Lohse B. (2005). How to Conduct a Cognitive Interview: A Nutrition Education Example.

[25] Jobe J.B., Mingay D.J. (1989). Cognitive research improves questionnaires. Am. J. Public Health.

[26] Drennan J. (2003). Cognitive interviewing: Verbal data in the design and pretesting of questionnaires. J. Adv. Nurs..

[27] Quandt S.A., Ip E.H., Saldana S., Arcury T.A. (2012). Comparing two questionnaires for eliciting cam use in a multi-ethnic us population of older adults. Eur. J. Integr. Med..

[28] Ernst E. (2006). Prevalence surveys: To be taken with a pinch of salt. Complement. Ther. Clin. Pract..

[29] Fonnebo V., Wiesener S., Falkenberg T., Hegyi G., Hök J., di Sarsina P.R. (2012). Legal Status and Regulation of CAM in Europe: Part I—CAM Regulations in the European Countries.

[30] Fonnebo V., Kristiansen T.T., Falkenberg T., Hegyi G., Hök J., di Sarsina P.R., Wiesener S. (2012). Legal Status and Regulation of CAM in Europe: Part II—Herbal and Homeopathic Medicinal Products.

[31] Fonnebo V., Wiesener S., Falkenberg T., Hegyi G., Hök J., di Sarsina P.R. (2012). Legal Status and Regulation of CAM in Europe: Part III—CAM Regulations in EU/ETFA/EEA.

